# Autosomal Dominant Gyrate Atrophy-Like Choroidal Dystrophy Revisited: 45 Years Follow-Up and Association with a Novel *C1QTNF5* Missense Variant

**DOI:** 10.3390/ijms22042089

**Published:** 2021-02-19

**Authors:** Ulrich Kellner, Nicole Weisschuh, Silke Weinitz, Ghazaleh Farmand, Sebastian Deutsch, Friederike Kortüm, Pascale Mazzola, Karin Schäferhoff, Valerio Marino, Daniele Dell’Orco

**Affiliations:** 1 Zentrum für Seltene Netzhauterkrankungen, AugenZentrum Siegburg, MVZ Augenärztliches Diagnostik- und Therapiecentrum Siegburg GmbH, Europaplatz 3, 53721 Siegburg, Germany; weinitz@augenzentrum-siegburg.de (S.W.); farmand@augenzentrum-siegburg.de (G.F.); deutsch.bastian@googlemail.com (S.D.); 2 RetinaScience, Postfach 301212, 53192 Bonn, Germany; 3 Center for Ophthalmology, Institute for Ophthalmic Research, University of Tübingen, 72076 Tübingen, Germany; nicole.weisschuh@uni-tuebingen.de; 4 Center for Ophthalmology, University Eye Hospital, University of Tübingen, 72076 Tübingen, Germany; friederike.kortuem@uni-tuebingen.de; 5 Institute of Medical Genetics and Applied Genomics, University of Tübingen, 72076 Tübingen, Germany; pascale.mazzola@med.uni-tuebingen.de (P.M.); karin.schaeferhoff@med.uni-tuebingen.de (K.S.); 6 Department of Neurosciences, Biomedicine and Movement Sciences, Section of Biological Chemistry, University of Verona, 37134 Verona, Italy; valerio.marino@univr.it (V.M.); daniele.dellorco@univr.it (D.D.)

**Keywords:** autosomal dominant gyrate atrophy-like choroidal dystrophy (adGALCD), late-onset retinal dystrophy (LORD), C1QTNF5, genetic modeling, long-term follow-up

## Abstract

We present a long-term follow-up in autosomal dominant gyrate atrophy-like choroidal dystrophy (adGALCD) and propose a possible genotype/phenotype correlation. Ophthalmic examination of six patients from two families revealed confluent areas of choroidal atrophy resembling gyrate atrophy, starting in the second decade of life. Progression continued centrally, reaching the fovea at about 60 years of age. Subretinal deposits, retinal pigmentation or choroidal neovascularization as seen in late-onset retinal degeneration (LORD) were not observed. Whole genome sequencing revealed a novel missense variant in the *C1QTNF5* gene (p.(Q180E)) which was found in heterozygous state in all affected subjects. Haplotype analysis showed that this variant found in both families is identical by descent. Three-dimensional modeling of the possible supramolecular assemblies of C1QTNF5 revealed that the p.(Q180E) variant led to the destabilization of protein tertiary and quaternary structures, affecting both the stability of the single protomer and the entire globular head, thus exerting detrimental effects on the formation of C1QTNF5 trimeric globular domains and their interaction. In conclusion, we propose that the p.(Q180E) variant causes a specific phenotype, adGALCD, that differs in multiple clinical aspects from LORD. Disruption of optimal cell-adhesion mechanisms is expected when analyzing the effects of the point mutation at the protein level.

## 1. Introduction

Inherited diffuse choroidal dystrophies with peripheral onset include two well defined disorders, gyrate atrophy (MIM 258870) and choroideremia (MIM 303100) [1,2]. Gyrate atrophy is an autosomal recessively inherited disorder associated with mutations in the *OAT* gene and hyperornithinemia [1]. Choroideremia is an x-linked inherited disorder associated with mutations in the *CHM* gene [2]. Several simplex cases and few families which are not sufficiently defined to form another entity have been reported. Of these, autosomal dominant disorders include retinitis pigmentosa with predominant choroidal atrophy associated with a dominant *RPE65* mutation [3] as well as a late-onset night blindness with trickle like macular dystrophy [4].

Autosomal dominant choroidal dystrophy has been rarely reported as diffuse choroidal dystrophy initially involving the posterior pole [5] and in few families with peripheral onset similar to gyrate atrophy. Some of the latter families were reported prior to the knowledge of the association of hyperornithinemia and gyrate atrophy [6]. One family was reported with exclusion of hyperornithinemia and the observed phenotype has been termed autosomal dominant gyrate atrophy-like choroidal dystrophy (adGALCD) [7].

**Figure 1:**
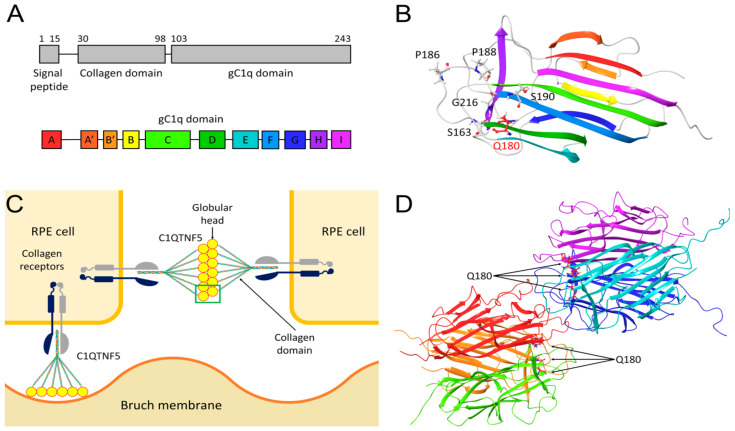
(**A**) Domain organization of C1QTNF5 (top) and secondary structure elements (bottom) with the coloring scheme and labeling of the individual β-strands. (**B**) The three-dimensional structure of a C1QTNF5 protomer is shown in cartoon, with β-strands colored according to panel A, residues S163, P186, P188, S190 and G216, whose mutations are late-onset retinal degeneration (LORD)-associated, are shown in grey sticks and labeled. Residue Q180, whose E variant is autosomal dominant gyrate atrophy-like choroidal dystrophy (adGALCD)-associated in this work, is represented in red sticks and labeled. N atoms are colored in blue, O atoms in red, H atoms in white. (**C**) Schematic representation of the C1QTNF5-mediated interactions of retinal pigment epithelium (RPE) cells with neighboring cells and with Bruch membrane. The collagen domain binds to collagen receptors on the membrane of an RPE cell, while the globular heads of the bouquet-like C1QTNF5 multimer can interact with the globular heads of another C1QTNF5 multimer bound to an adjacent RPE cell or with Bruch membrane. The structure of the two globular heads framed in green is represented in panel D. (**D**) Three-dimensional structure of the two trimers constituting the globular heads of C1QTNF5 belonging to different RPE cells and involved in cell adhesion based on available X-ray crystallographic data. Protein structure is shown in cartoon, chain A of is colored in red, chain B in orange, chain C in green, chains A, B and C of the second molecule are represented in cyan, blue and purple, respectively. Residues Q180 are shown as red sticks and labeled.

Late-onset retinal degeneration (MIM 605670; LORD) is an autosomal dominantly inherited disorder with several distinct clinical signs, which include initially mid-peripheral temporal subretinal deposits developing into scalloped choroidal atrophy and progression towards the macula with choroidal neovascularization in later stages [8,9,10]. Most cases with LORD have been associated with the p.(S163R) variant in the *C1QTNF5* gene, indicating that this particular variant is a founder mutation [11]. Few additional LORD families with different variants in the *C1QTNF5* gene have been reported, including the recurrent p.(S163R) variant as well as additional missense variants (p.(P188T), p.(G216C), p.(P186S) and p.(S190W)) located in the gC1q domain of the C1QTNF5 protein [12,13] (Figure 1A,B).

The human *C1QTNF5* gene encodes for a 25 kDa secretory protein expressed in adipose tissue [14], ciliary body of the eye and in the retinal pigment epithelium (RPE) [15]. C1QTNF5 is composed by three domains, namely the signal peptide (residues 1–15), the collagen domain (residues 30–98) and the gC1q domain (residues 103–243) [11,16] (Figure 1A). Specifically, the collagen domain allows the association with the plasma membrane via collagen receptors, while the gC1q domain is responsible for RPE cell adhesion with other RPE cells and with Bruch membrane [17,18] (Figure 1C). As a member of the C1q family [19], C1QTNF5 protomers assemble into trimers due to the intertwining of their collagen domains. The association of the collagen domains brings the gC1q domains close to one another, thus favoring the packing of the hydrophobic “zipper” box at the protomer–protomer interface, ultimately leading to the constitution of a globular domain (or globular head). Additionally, six globular trimers furtherly assemble into a bouquet-like octadecameric multimer [18] via the association of their collagen triple helices (Figure 1C,D).

The hydrophobic plateau constituted by residues 181–186 of each protomer [16] gives rise to an extended hydrophobic surface responsible for the binding of an RPE cell [15] to a neighboring cell or to the underlying Bruch membrane [18].

The purpose of the present study was to present long-term follow-up of the original adGALCD family [7] and of a distantly related second family to better characterize the clinical course and the retinal morphology and function in this disorder. In addition, we report an association of adGALCD with a novel *C1QTNF5* missense variant and discuss possible genotype/phenotype correlation based on in silico molecular analysis.

## 2. Results

### 2.1. Clinical Evaluation of Family Members

**Figure 2:**
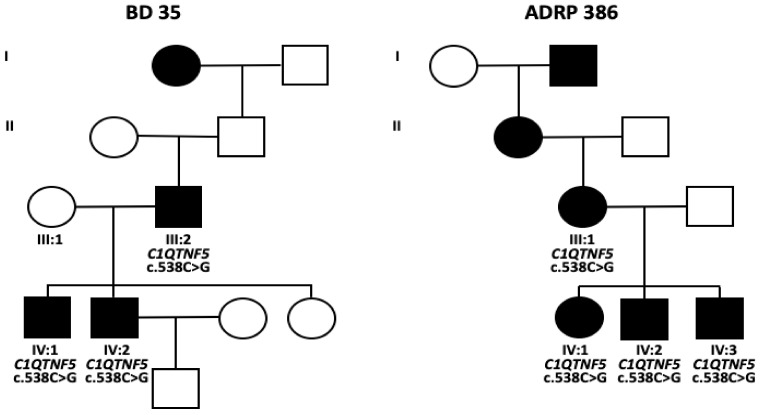
Pedigrees and genotypes of both families. I–IV: consecutive generations.

Two families were included in this study. For family BD 35 with three affected males, a father and his two sons, initial findings have been reported previously [7]. Ornithine blood levels were normal in all three patients. The mother of both sons (III:1) had normal findings at 68 years of age. The parents of patient III:2 died prior to 45 years of age without reported eye problems. The maternal grandmother of patient III:2 was reported to be affected. For one of the sons (IV:2), regular follow-up visits were undertaken between 38 and 63 years of age. In family ADRP 386, three affected siblings (IV:1, IV:2 and IV:3) were examined between 50 to 58 years of age. Further family members in three previous generations were reported to be affected (Figure 2) but were not available for clinical examination.

Onset of disease with peripheral atrophic lesions was documented in patient IV:2 from family BD 35 at 18 years of age and in patient IV:2 from family ADRP 386 at 24 years of age (Table 1). Functional problems were noted later at about 40 years of age (patients III:2 and IV:1 from family BD35; patients IV:2 and IV:3 from family ADRP 386) with the onset of night blindness. In the same patients, subjective problems due to visual field defects were noted at about 50 years of age.

Snellen visual acuity ranged between 0.5 to 1.0 in less progressed stages and was reduced to 0.1 to 0.4 when the residual visual field was smaller than 10 degrees (Table 1). Refractive errors were most mildly hyperopic with moderate astigmatism.

Cataract surgery was performed in one of the patients by one of the authors (U.K.) without complications. In this patient (IV:2 from family BD 35) as well as in all other patients without prior cataract surgery no long anterior lens zonules were noted (Table 2).

**Figure 3:**
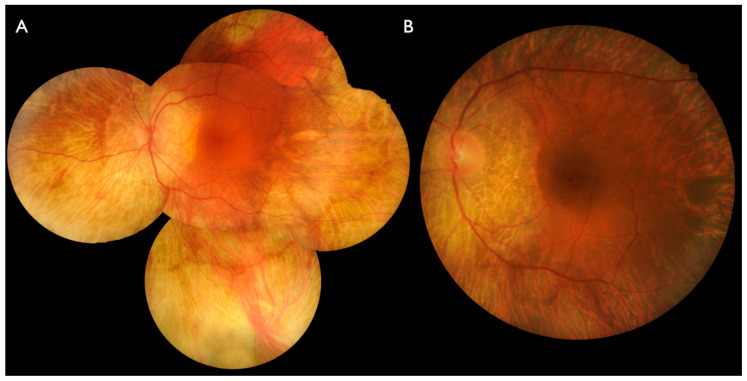
Fundus photography in family ADRP 386 in patient IV:2 at 53 years of age (**A**) and patient IV:3 at 56 years of age (**B**). Peripapillary atrophy is present in both patients. Peripheral confluent atrophic areas have approached to the macular area except from superior in patient IV:2, whereas circular atrophy up to the vascular arcades is present in patient IV:3.

**Figure 4:**
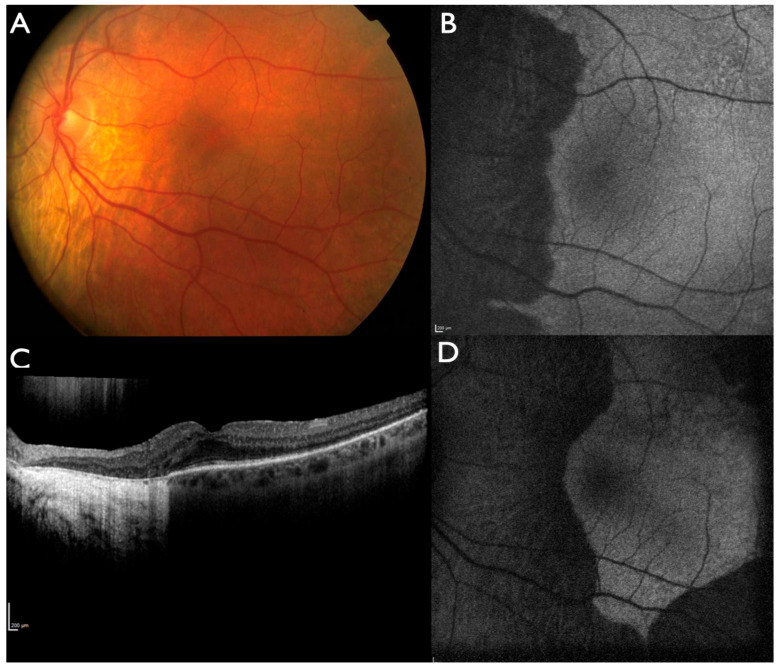
Progression of choroidal atrophy in patient IV:2 from family BD 35. Fundus photography at 38 years of age (**A**). Fundus autofluorescence at 51 years of age (**B**). Optical coherence tomography (OCT) (**C**) and fundus autofluorescence at 63 years of age (**D**). The progression of peripapillary atrophy towards the fovea is slow, whereas peripheral lesions progressed towards the posterior pole at age 63.

Retinal findings started with peripheral and peripapillary chorioretinal atrophic lesions. The lesions presented as large atrophic areas which were sharply demarcated by small areas of preserved retinal pigment epithelium (RPE) between lesions (Figure 3 and Figure 4). Thus, the findings resembled more the appearance of gyrate atrophy than choroideremia. During progression, the peripheral lesions circumferentially encroached towards the fovea, reaching first the inferior vascular arcades latest at 50 years of age (Figure 3 and Figure 4). In contrast, the progression of the peripapillary atrophy from the disc towards the fovea was slow (Figure 4). In late stages (patients III:2 from family BD 35 and IV:3 from family ADRP 386) only the foveal area was preserved. Pigmented lesions were either absent or minimal.

**Figure 5:**
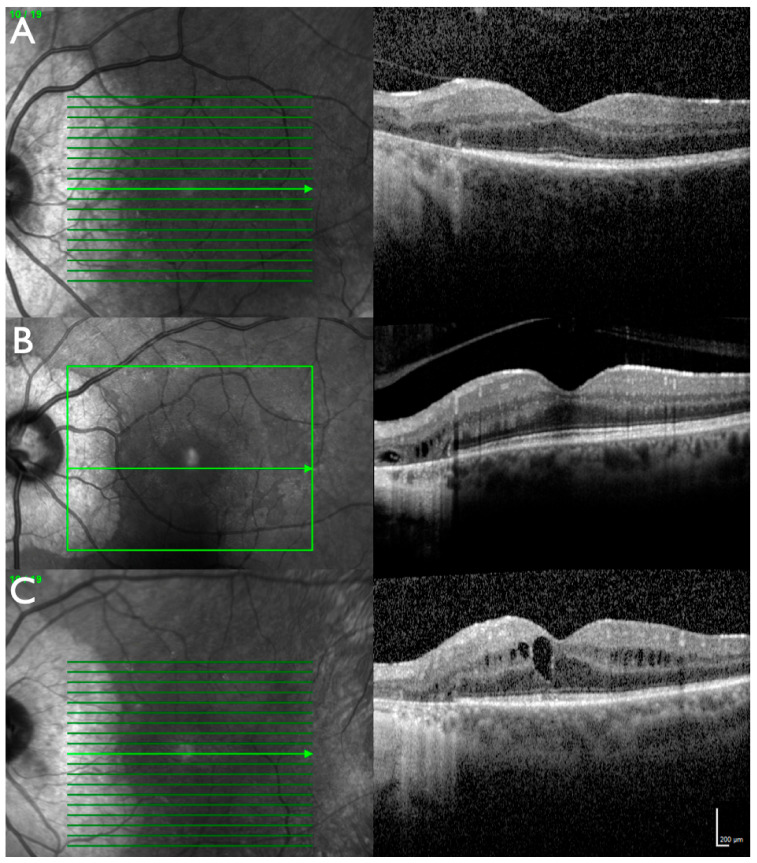
OCT of family ADRP 386 (patient IV:2 (**A**), patient IV:1 (**B**), patient IV:3 (**C**)). Peripapillary atrophy is associated with loss of retinal pigment epithelium and outer retinal layers. Those layers appear normal in the areas without atrophy, the border is sharply demarcated. Patient IV:1 shows some intraretinal fluid in the atrophic area, whereas patient IV:3 presents with cystoid macular edema.

**Figure 6:**
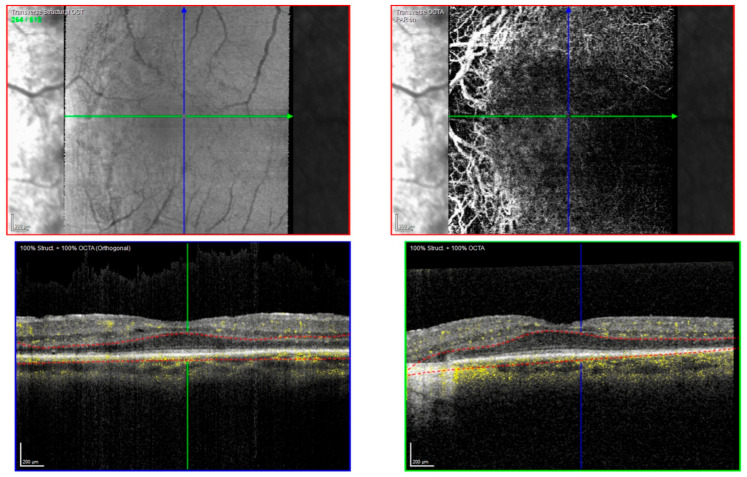
OCT-angiography on the left eye of patient IV:2 from family BD 35 with OCT-C-Scan (upper left), OCT angiography (upper right) and corresponding OCT-B-Scans below. Marked choriocapillaris and choroidal vessel atrophy is present in the atrophic areas.

Fundus autofluorescence (FAF), near-infrared autofluorescence (NIA) and optical coherence tomography (OCT) were normal in the non-affected regions with a sharp border towards the affected areas (Figure 4 and Figure 5). Affected areas showed absence of FAF, NIA and a complete loss of outer retinal structures in the OCT indicating loss of photoreceptors and RPE. OCT-angiography showed absence of choriocapillaris and few remaining large choroidal vessels with reduced flow in the affected areas (patient IV:2 from family BD 35, Figure 6). Progression of disease could be documented over time (Figure 4). Only in patient IV:3 from family ADRP 386 cystoid macular edema was observed (Figure 5).

**Figure 7:**
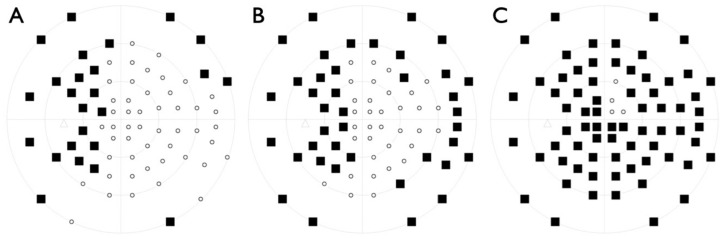
Visual field progression in patient IV:2 from family BD 35 between (**A**) 51, (**B**) 56 and (**C**) 60 years of age.

**Figure 8:**
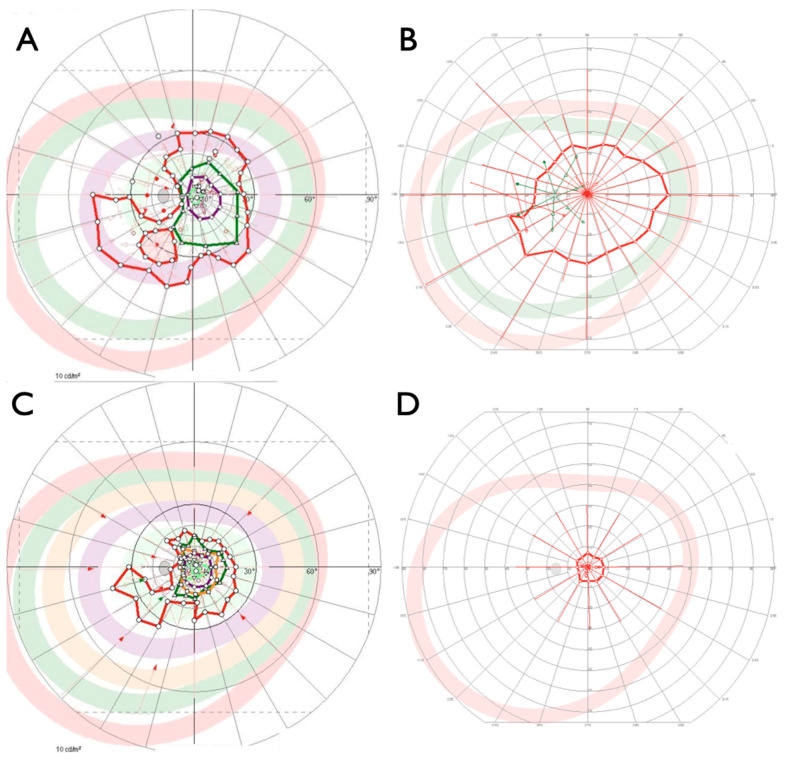
Visual fields in patient IV:1 (**A**), patient IV:2 (**B**) and patient IV:3 from family ADRP 386 at 50 (**C**) and 56 years of age (**D**). Please note that visual fields were obtained with different software and therefore cannot be displayed with the same look. Degrees are scaled similarly for all images.

Visual fields were variably constricted corresponding to the chorioretinal atrophic lesions and scotomata progressed similarly over time (Figure 7 and Figure 8).

In patient IV:2 from family BD 35, full-field ERG was reported as slightly abnormal with an unspecified method at 18 years of age and the EOG showed a severely reduced light rise. At 38 and 42 years of age, ERG amplitudes according to ISCEV standards were markedly reduced. At 50 years of age, only residual responses were recordable in the ERG. Similarly, no measurable or small residual responses could be recorded in the father (patient III:2) as well as in all patients of family ADRP 386 between 50 to 58 years of age. The mfERG, recorded in three patients, showed centrally preserved responses with reduced amplitude but normal implicit time corresponding to areas with preserved retina and absent responses in atrophic areas.

### 2.2. Identification of a Novel Missense Variant in C1QTNF5

The self-reported family history of family BD 35 was indicative of an autosomal dominant pattern of inheritance for family BD 35 while it was consistent with an autosomal dominant inheritance mode for family ADRP 386 with affected individuals in four consecutive generations.

Patients III:2 and IV:2 from family BD 35 as well as patients IV:1 and IV:3 from family ADRP 386 underwent diagnostic genetic testing by whole genome sequencing. Putative pathogenic variants were only identified in the *C1QTNF5* gene, namely a novel missense variant (c.538C > G/p.(Q180E)), which was found in heterozygous state in all family members that were available for genetic testing (Figure 2). The variant is absent from the population database gnomAD (https://gnomad.broadinstitute.org/), indicating that it is very rare. In silico predictions of the p.(Q180E) variant using various online tools were discordant. It was predicted to be possibly damaging (PolyPhen2), disease causing (MutationTaster), neutral (PROVEAN) or tolerated (SIFT) (Appendix A Table A1). Sequence alignment of *C1QTNF5* and orthologous proteins in other species showed that p.(Q180E) is fully conserved in 176 vertebrate species (Appendix A Table A2). Accordingly, the high level of evolutionary conservation is reflected by high scores obtained with PhyloP, CADD and FATHMM (Appendix A Table A1).

**Figure A1:**
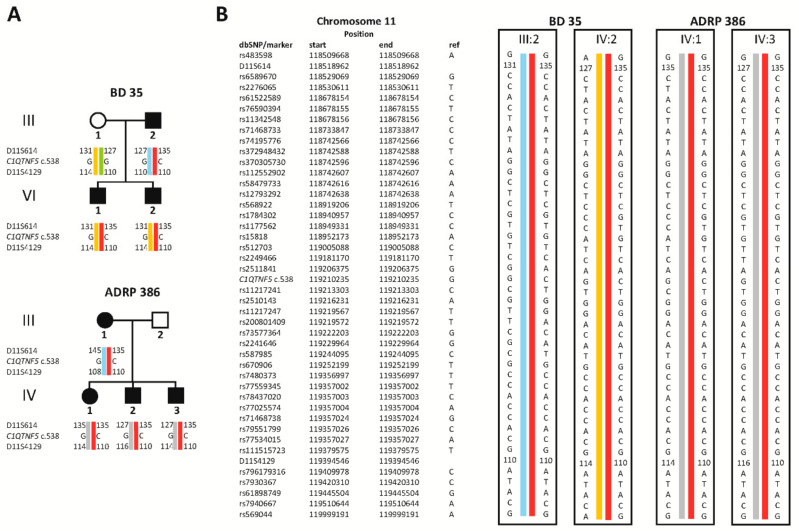
Haplotype analysis showing that the p.(Q180E) variant found in families BD 35 and ADRP 386 is identical by descent. (**A**) Pedigrees showing the core families. Genotypes of the *C1QTNF5* variant and two microsatellites surrounding the gene are depicted below the respective pedigree symbols. The shared haplotype of the p.(Q180E) variant is indicated by red colored bars. (**B**) Extended haplotype analysis using informative genotype data of 41 SNPs spanning a physical region of 2 Mb interval surrounding *C1QTNF5*. Note that SNP genotypes were deduced from whole genome sequencing data that was only available for individuals III:2 and IV:2 from family BD 35, and for individuals IV:1 and IV:3 from family ADRP 386, respectively. The shared haplotype of the p.(Q180E) variant is indicated by red colored bars. Genomic coordinates refer to GRCh37/hg19. Ref, reference allele.

As far as could be established from the self-reported family histories the two families are either not or very distantly related (i.e., not being aware of their relatedness). In order to distinguish between the alternative hypotheses of recurrent mutation and identity by descent, we established haplotypes using SNP data obtained by whole genome sequencing. In addition, two annotated microsatellites in the vicinity of *C1QTNF5* were analyzed. Appendix A Figure A1 shows that p.(Q180E) is associated with the same haplotype on all disease alleles analyzed. This is consistent with the mutant allele being identical by descent.

Patients of both families presented with similar clinical findings and segregated the same variant. This suggested an association with the novel p.(Q180E) variant with adGALCD.

### 2.3. Molecular Modeling of C1QTNF5 and Analysis of the Structural and Functional Effects of the Missense Mutation p.(Q180E)

The autosomal dominant nature of adGALCD and LORD implies that, in the pool of expressed C1QTNF5 proteins, half of the molecules will carry the Q180E mutation, whereas the other half will be unaffected (wild-type, WT). This fact, together with the supramolecular organization of C1QTNF5 (Figure 1) opens up a very complex scenario, in which both WT and mutated protomers can form assemblies, whose protomers can either be mutated or not. We considered all these possibilities in our molecular modeling analyses.

The Q180E amino acid substitution could in first instance perturb the stability of each C1QTNF5 protomer. To evaluate such a possibility, Gln180 was replaced with the negatively charged Glu. The apparent change in free energy of folding with respect to the WT (∆∆G_f_^app^) was calculated for the single protomer, resulting in a 2.56 kcal/mol destabilization. Replacement of superficial protein charges may perturb long-range electrostatics in proteins, with significant effects on protein stability [20]. A first effect of the Q180E mutation could therefore be the destabilization of the tertiary structure of C1QTNF5.

**Figure 9:**
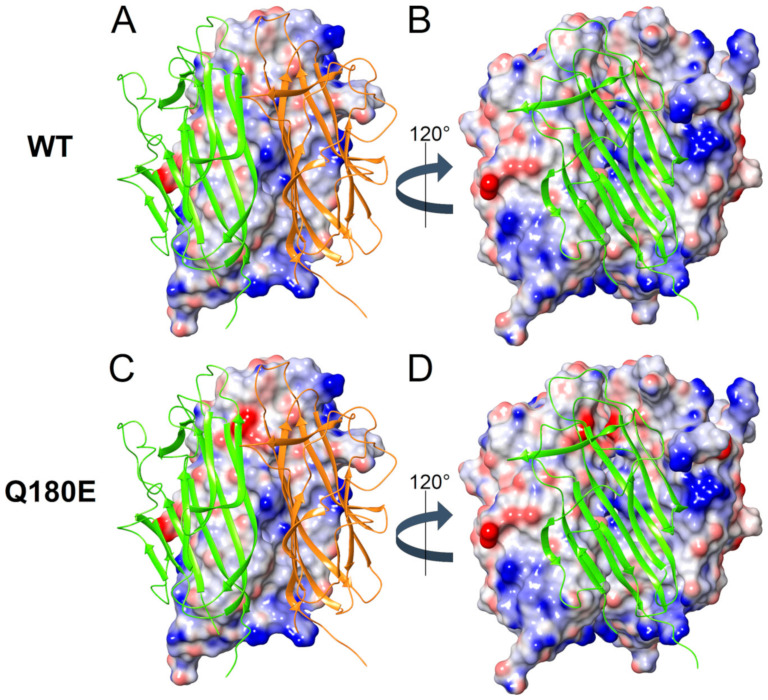
Electrostatic potential mapped on the molecular surface of a C1QTNF5 wild-type (WT) (**A**,**B**) and Q180E (**C**,**D**) homozygous trimer. Protein structure is shown in cartoon, protomer B is colored in orange, protomer C in green. The molecular surface of protomers A and B is colored in a blue-to-red scale from −0.3 to 0.3 kT/e. (**A**,**C**) Electrostatic potential of protomer A. (**B**,**C**) Electrostatic potential of protomers A and B, protein view is rotated by 120 ° counterclockwise along the symmetry axis.

Residue Q180 is the last residue of β-strand E (Figure 1B) and it is located between the hydrophobic plateau and the hydrophobic “zipper” box. The analysis of the protomer surface highlighted that residue Q180 is fundamental for the reciprocal interaction among the three protomers constituting the globular head of C1QTNF5. Indeed, the aggregation score provided by AggScore (11052) for residue Q180 resulted to be the highest value reported for the entire protein sequence, suggesting that mutations involving Q180 may have a detrimental effect on the association of the trimeric globular head and its stability. Considering the whole globular head, the substitution of a polar uncharged residue with a negatively charged residue close to the highly hydrophobic “zipper” interface altered the partial charge distribution and therefore the electrostatic potential on the protomer surface (Figure 9A,C). Such alteration resulted in an electrostatic repulsion between the negative charges of two interacting protomers (Figure 9B,D), affecting both the stability of the complex (∆∆G_f_^app^ = 30.5 ± 1.0 kcal/mol, Table 3) and the affinity of the protomers for one another ∆∆G_b_^app^ = 12.2 ± 5.5 kcal/mol, Table 3). On the other hand, the presence of two or even three mutations within the same trimer (Figure 9B,D) further increased the electrostatic repulsion, thus causing a destabilization of the complex (∆∆G_f_^app^ = 64.8 ± 1.6 and 101.12 ± 0.04 kcal/mol, respectively, Table 3) and a decrease in affinity of the protomers (∆∆G_b_^app^ = 27.7 ± 6.6 and 44.7 ± 1.4 kcal/mol, respectively, Table 3). It is worth noting that the contribution of coulombic repulsion to the apparent binding free energy among protomers in the trimer impacted both affinity and stability. On the contrary, the contribution of solvation of the mutated trimer decreased the apparent affinity of the protomers (∆∆G_b_^app^ S = 9.4 ± 1.2 kcal/mol, Table 4) but it was found to increase the stability of the ternary complex (∆∆G_f_^app^ S = −45.5 ± 1.2 kcal/mol, Table 4).

**Figure 10:**
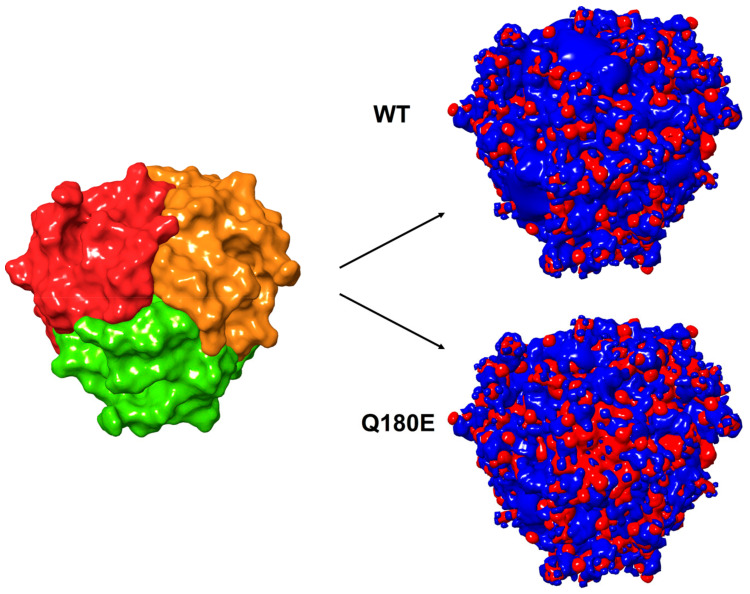
Electrostatic isopotential surfaces of WT and Q180E homozygous C1QTNF5 trimers forming a single globular head show unfavorable electrostatic contributions to association in the case of mutated protomers. The top view of the molecular surface of protomers A, B and C (colored in red, orange and green, respectively) is shown to clarify the relative orientation of each protomer. The electrostatic isopotential surfaces corresponding to −10 kT/e and 10 kT/e are shown in red and blue, respectively.

In summary, the Q180E substitution was predicted to generate a significant electrostatic repulsion among the protomers forming up a globular head, whose effects could propagate at longer distance, considering the significantly changed electrostatic potential generated by a trimer (Figure 10), which could affect the recognition between two adjacent globular heads approaching each other. Indeed, on a larger scale, the Gln-to-Glu substitution in position 180 also affected the interaction between adjacent globular heads belonging to different cells, as shown by the positive ∆∆G_b_^app^ and ∆∆G_f_^app^ shown by the mutants (Table 3). Similar to the interaction among protomers of the same globular head (Figure 10), the top-to-top interaction between globular heads was increasingly destabilized by the Q180E mutation proportionally to the number of mutations present in the two trimers. Indeed, the remapping of electrostatic potential involving globular heads’ interface resulted in an increase of ∆∆G_b_^app^ from 9.0 kcal/mol in the presence of 1 protomer carrying the mutation to approximately 60 kcal/mol in the presence of the homozygous variant (Table 3), while ∆∆G_f_^app^ increased from 26.7 to 187.8 kcal/mol under the same conditions (Table 3). Interestingly, the presence of mutations was favorable in terms of electrostatic interactions between the two globular heads from both the affinity and stability standpoints, as shown by the negative ∆∆G values for both binding and folding (∆∆G_b_^app^ C and ∆∆G_f_^app^ C columns, Table 3). On the other hand, such positive coulombic contribution was counterbalanced by the larger, unfavorable increase in ∆∆G of solvation (∆∆G_b_^app^ S and ∆∆G_f_^app^ S columns, Table 3) resulting from the addition of a negatively charged residue to a highly hydrophobic patch.

## 3. Discussion

Autosomal dominant gyrate atrophy-like choroidal dystrophy (adGALCD) appears to be a distinct entity that has been described so far in three families. Two of them are included in this manuscript, while images consistent with the ocular phenotype have already been reported for another family without detailed information [6]. As all three families were observed in Germany, it might well be that they are all related. This assumption is supported by haplotype analysis for the two families reported in the present study.

Compared to the clearly defined entities of gyrate atrophy and choroideremia, the onset is later and the rate of progression in adGALCD is slower, e.g., reading ability may be preserved up to 70 years of age. The chorioretinal lesions are more clearly demarcated compared to choroideremia. In contrast to gyrate atrophy, early onset of cataract formation and hyperornithinemia were not observed, and cystoid macular edema was only seen in one patient.

The finding of a putative pathogenic variant in the *C1QTNF5* gene supports the inclusion of adGALCD into the spectrum of LORD. However, the clinical findings differ in several aspects between adGALCD and LORD (Table 4). The onset of retinal alterations is earlier and more peripheral in adGALCD compared to LORD [21,22,23,24,25,26]. In contrast to adGALCD with peripheral retinal changes present at least at the end of the second decade of life, patients with stage 1 LORD are reported to show a normal retina until 40 years of age, while long anterior lens zonules and iris atrophy may already by present [26,27]. The peripheral lesions in adGALCD are associated with early peripheral visual field loss, while the peripheral visual fields remain intact in LORD even in progressed stages [25]. Characteristic findings of stage 2 LORD are subretinal deposits which present in a pseudodrusen-like pattern with sub-RPE material and a thickening between RPE and Bruch membrane in the OCT [9,11,21,22,23,28,29,30]. These pseudodrusen-like lesions first develop in the temporal middle periphery progressing to scalloped chorioretinal atrophy as well as pigmentary changes, all of which were not observed in the adGALCD families. Although scalloped chorioretinal atrophic lesions have been observed in adGALCD and LORD [4,9,13,21,22,23,24,25,31,32,33,34], scalloped lesions in LORD are located in the mid-periphery and are much smaller in size compared to those observed in the patients in the present and previous study [7]. Stage 3 LORD is characterized by atrophic macular alterations or choroidal neovascularizations [11,24,27,34,35], whereas in adGALCD neovascular lesions were not observed and the fovea was preserved until the final stage. As such, the phenotype in the families presented in this study much more resembles gyrate atrophy than LORD. Distinct characteristics of LORD in FAF and OCT were not observed in adGALCD: no sub-RPE deposits in the OCT and no structural alterations prior to RPE loss in the FAF were detected [25,26,29,33,36].

In addition, retinal function was earlier and more severely affected in adGALCD as indicated by earlier and markedly reduced ERG and multifocal ERG responses. Normal or well-preserved ERG has been reported up to 60–67 years of age in LORD [23,33,37].

A frequent additional finding in LORD are long anterior lens zonules [12,22,24,25,28,32,38,39], although they are not present in all LORD patients [13] and may also occur in the absence of LORD [40]. Long anterior lens zonules were not observed in the patients in this study. In addition, no problems during cataract surgery were observed or reported in contrast to LORD [41].

A similarity between adGALCD and LORD is the development of night blindness at about 40 years of age in most patients [26]. This appears unexplained for both disorders, while at that age still sufficient rod-rich areas along the vascular arcades appear to be structurally normal in the patients presented here as well as in LORD. As previously suggested [26], *C1QTNF5* gene variants may affect rod function with an additional mechanism which still has to be defined.

The *C1QTNF5* gene product is present in the lateral and basal membrane of retinal pigment epithelial cells and the ciliary body [42]. It interacts with HTRA1 in mice and affects extracellular matrix turnover, which might explain the development of subretinal deposits in LORD [43,44,45]. Following the resolution of X-ray structures of the globular domain of C1QTNF5 [16,18], molecular models were built to explain the mechanism of C1QTNF5-mediated cell adhesion among RPE cells and RPE-Bruch membrane. The typical bouquet-like octadecameric organization of C1QTNF5 ensuring the appropriate scaffold for cell-cell communication can be reached only by specific molecular recognition between C1QTNF5 subunits within the same and with surrounding globular head domains. Point mutations of key amino acids involved in the protein–protein recognition process can therefore lead to destabilization of these supramolecular assemblies. In case of autosomal dominant diseases, homo- and hetero-complexes can be formed by the expressed WT and mutant proteins, thus generating a complex molecular scenario that has emerged recently, with specific effects depending on the nature of the complex [46,47]. In the case of C1QTNF5, the scenario is extremely complex, with up to 64 possible assemblies between wild-type and individually mutated protomers. As a general conclusion, our modeling study predicts that, independently on the number of mutated protomers, the p.(Q180E) substitution will perturb both the stability of the globular heads and the recognition between adjacent trimers. This would result in a perturbation of the molecular scaffold linking to adjacent cells, ultimately disrupting the native cell-adhesion mechanisms.

## 4. Materials and Methods

### 4.1. Patient Enrollment and Retrieval of Blood Samples

Patients were recruited and clinically examined either at the Eye Hospital of the University of Tübingen (family ADRP 386) or at the AugenZentrum Siegburg (family BD 35). Genomic DNA of patients was extracted from peripheral blood using standard protocols. Samples from all patients were recruited in accordance with the principles of the Declaration of Helsinki and were obtained with written informed consent accompanying the patients’ samples. The study was approved by the institutional review board of the Ethics Committee of the University Hospital of Tübingen under the study numbers 349/2003V and 116/2015BO2.

### 4.2. Clinical Examination

Family BD 35 included four members who have been examined, three of them affected males including the father and both of his sons (see pedigree depicted in Figure 2). Details of these examinations have been reported previously [7]. The father (III:2) was lost to follow-up due to stroke-associated death and one son (IV:2) due to a brain tumor. The second son (IV:2) was re-examined at different time intervals between 38 to 63 years of age. In addition to basic ophthalmologic examination, he underwent visual field testing, full-field electroretinography (ERG) and multifocal ERG (mfERG) recording, wide-angle fundus autofluorescence (FAF), near-infrared autofluorescence (NIA), spectral domain OCT (OCT) examinations and OCT-angiography. Family ADRP 386 comprises three siblings. All three siblings underwent in addition to basic ophthalmologic examination visual field testing, ERG and mfERG recording, FAF and OCT examination. Please note that the phenotype in family ADRP 386 was initially described as autosomal dominant retinitis pigmentosa (ADRP) but that the original family ID was kept after refinement of the clinical diagnosis based on examination of more family members and longer follow-up.

Electrophysiologic examinations and non-invasive retinal imaging were obtained as described previously after medical dilatation of the pupil [48,49]. Full-field ERG and mfERG were measured according to the most recent ISCEV standards [50,51] at the time of recording. FAF and NIA were obtained with a confocal scanning laser ophthalmoscope (Heidelberg Retina Angiograph 2, Heidelberg Engineering, Heidelberg, Germany) using 30° and 50° lenses. Volume and single scan SD-OCT as well as OCT-angiography were performed with a Spectralis OCT (Heidelberg Engineering, Heidelberg, Germany).

### 4.3. Diagnostic Genetic Testing

Patients III:2 and IV:2 from family BD 35 and patients IV:1 and IV:3 from family ADRP 386 underwent diagnostic genetic testing by means of whole genome sequencing. Details of sequencing and variant classification have been described previously [52]. Segregation analysis in family members was performed using conventional Sanger sequencing.

### 4.4. Haplotype Analysis

Haplotype analysis was performed in all available family members by genotyping two microsatellites in the vicinity of *C1QTNF5* (D11S614 and D11S4129). Primers for microsatellite amplification were as follows: D11S614-forward: 5′-ACAGACCCACCAGGACTAT-3′, D11S614-reverse: 5′-CCCGGATGTCTGCAAGGTGG-3′; D11S4129-forward: 5′-ACAGCGACCACATCTCCTGC-3′, D11S4129-reverse: 5′-GGCCACTGCCCTTACCATCA-3′. Genotyping of microsatellites was performed as described previously [53]. Extended haplotype analysis was performed in patients III:2 and IV:2 from family BD 35 and patients IV:1 and IV:3 from family ADRP 386 using informative genotype data of 41 SNPs spanning a physical region of 2 Mb interval surrounding *C1QTNF5*. Genotypes were considered informative if they could be unambiguously assigned to a haplotype. The genotype data were obtained from the whole genome sequencing datasets.

### 4.5. In Silico Predictions of Pathogenicity

Online prediction tools PolyPhen2 (http://genetics.bwh.harvard.edu/pph2/), MutationTaster (http://www.mutationtaster.org/), and PROVEAN and SIFT (http://provean.jcvi.org/genome_submit_2.php), were used to predict the impact of the p.(Q180E) substitution on *C1QTNF5*. PhyloP, CADD and FATHMM-MKL scores were retrieved from the megSAP pipeline (https://github.com/imgag/megSAP). Orthologous gene sequences were downloaded from NCBI (https://www.ncbi.nlm.nih.gov/), and amino acid sequences aligned using ClustalW2 (https://www.ebi.ac.uk/Tools/msa/clustalw2/).

### 4.6. Molecular Modeling of C1QTNF5 Protein Structure and Analysis of the p.(Q180E) Effects on Stability and Affinity

All molecular modeling analyses were performed within the environment of the chemical simulation software Maestro/Bioluminate (Schroedinger, New York, USA). Human C1QTNF5 trimer was modeled using as the starting structure the PDB file with entry 4F3J [16], which provided the highest resolution (1.34 Å) among the available structures, as well as the symmetric operators for reconstituting the trimeric biological unit. The hexameric assemble of two globular heads was built superimposing the trimers to the top-to-top interacting protomers of PDB file 4NN0 (resolution 1.42 Å) [18]. The trimeric globular heads were reconstituted using the symmetric operators previously included in PDB file 4F3J [16]. This allowed us to evaluate the effects of the pathogenetic point mutation within each individual protomer making up a globular head (trimer), as well as a couple of globular heads from two different cells, interacting with each other (interacting trimers, Figure 1C,D).

Protein structures were prepared according to the pipeline of the *Protein Preparation* tool, briefly consisting of the assignment of bond orders using Chemical Components Dictionary database (www.pdb.org, wwPDB Foundation, Piscataway NJ, USA), addition of H atoms, selection of the most probable rotamer and deletion of water molecules closer than 3.5 Å only to other water molecules. Structure refinement included sampling of water orientation, usage of crystal symmetry to optimize H-bonds with the other protomers and prediction of the protonation states of ionizable residues at pH 7.5 by PROPKA prior to H-bond assignment and optimization. Finally, protein structure was minimized using the OPLS3e forcefield (Schroedinger, New York, NY, USA) until the Root-Mean Square Displacement of the heavy-atom reached 0.3 Å.

The role of residue Gln180 (in the WT protein) in protomer association was investigated by the “Protein Surface Analysis” tool. A well-performing index based on the analysis of the distribution of hydrophobic and electrostatic patches on the surface of the protein, the AggScore index [54], was calculated to estimate aggregation propensity of the protomer.

The electrostatic potential surface was calculated by the “Poisson–Boltzmann Electrostatic Potential Surface” tool, by setting solute dielectric constant to 1, solvent dielectric constant to 80, solvent radius to 1.4 Å, temperature to 298 K, grid extension to 5 Å.

The “Residue Scanning” tool was employed to introduce the p.(Q180E) mutation in any combination of both the three protomers constituting a globular domain and the two interacting globular domains, thus resulting in 7 and 63 combinations, respectively. Each mutagenized structure was subjected to automatic selection of the most probable rotamer and energy minimization using the same parameters as those employed for the wild-type (WT) model.

The Molecular Mechanics/Generalized Born and Surface Area Continuum Solvation (MM/GBSA) method was used to predict the effects of residue mutation on protomer stability and affinity, in terms of relative changes compared to the WT, by using the specific thermodynamic cycle. It should be noticed that the free energy computed by the method is based on the MM force field without explicit contributions from conformational changes, therefore the obtained free energy values (∆∆G^app^ in Table 2, expressed in kcal/mol) should be taken as “apparent” values and considered as approximate indexes, which are useful in comparisons, rather than rigorously defined thermodynamic quantities. Protomer–protomer affinity was calculated for each of the three protomers against the other two protomers. The resulting 21 combinations were pooled for the number of mutations present in the trimeric assembly and the variations in the apparent binding affinity (∆∆G_b_^app^) and apparent stabilities (∆∆G_f_^app^). Presented in Table 3 are the average ± standard deviation of the respective group, whose size is also reported. Trimer–trimer affinity was calculated between the two globular heads, the 63 combinations obtained by “Residue Scanning” were grouped by the number of variants in the hexameric assembly with the same approach as that employed for protomer–protomer affinity. The contribution of electrostatics and solvation to the relative ∆∆G^app^ values for both binding and stability was specifically reported in Table 3 and discussed.

## Figures and Tables

**Table 1:** Patient data: examination, age of onset, refraction.

ID	Age First Exam	Age Last Exam	Follow-Up in Years	Age of Onset	Eye	Refraction First Exam	VA First Exam	Refraction Last Exam	VA Last Exam
**BD 35**									
III:2	40	70	30	40 NB	OD	NA	1.0	+1.0	0.16
					OS	NA	1.0	+0.50 −1.25/64°	0.16
IV:1	41	-	0	40 NB	OD	NA	1.0	-	-
					OS	NA	1.0	-	-
IV:2	18	63	45	18 RA, VFD	OD	+2.25 −2.25/11°	1.0	−0.50 −0.75/75°	0.63
					OS	+2.25 −1.75/172°	1.0	+3.25 −1.25/166°	0.63
**ADRP 386**									
IV:1	58	-	0		OD	−0.25 −2.25/168°	0.5	-	-
					OS	±0.00 −0.50/25°	0.63	-	-
IV:2	53	-	0	24 RA 39 NB 53 VFD, P	OD	+1.75 −1.50/94°	1.0	-	-
					OS	+1.50 −1.50/95°	1.0	-	-
IV:3	50	56	6	40 NB 50 VFD, AP, P	OD	−0.75 −1.75/90°	0.63	−0.75 −2.50/87°	0.4
					OS	−0.25 −1.50/85°	0.63	−1.75 −1.50/95°	0.25

AP: adaptation problems, NB: night blindness, P: photophobia, RA: retinal alterations, VA: visual acuity, VFD: visual field defects, OD: right eye, OS: left eye.

**Table 2:** Patient data: Anterior segment, retinal and functional findings.

ID	Anterior Segment	Retina	Optic Disc	ERG	Dark Adaptation	mfERG	Color Vision
***BD 35***							
III:2	IOL OU (63)	Residual foveal island, no pigmentation	pale	No residual response (52/62)	ND	ND	ND
IV:1	normal	Peripheral and peripapillary atrophic lesions, no pigmentation	vital	ND	ND	ND	ND
IV:2	normal, IOL OD (63)	Progressive peripheral and peripapillary atrophic lesions, no pigmentation	vital	slightly abnormal (18); Severely reduced (42); No residual responses (54)	Mildly increased threshold (38)	Centrally preserved but reduced responses, normal implicit time (54)	Nagel anomaloscope: normal; PD15 desaturated: minor errors
***ADRP 386***							
IV:1	IOL OU (54)	Peripheral and peripapillary atrophy, no pigmentation	vital	Minimal photopic residual response	Increased threshold for white, red, blue	ND	ND
IV:2	normal	Peripheral and peripapillary atrophy, Minimal pigmentation OCT: OD mild ERM	vital	Residual responses	ND	Centrally preserved but reduced, normal implicit time	PD15 desaturated and saturated: minor errors
IV:3	normal	Progressive peripheral and peripapillary atrophic lesions, limited pigmentation, cystoid macular edema	vital	No residual responses (50)	Markedly increased threshold for white, red, blue (56)	Centrally preserved but reduced responses, normal implicit time (50)	PD15 desaturated and saturated: minor errors (56)

ERG: full-field electroretinography, mfERG multifocal ERG, IOL: intraocular lens, OU: both eyes, OD: right eye, ND: not done. For the three patients examined more than once, numbers in brackets indicate age of patient at the time of the examination.

**Table 3:** Effects of the number of Q180E-mutated protomers on the relative affinity (∆∆G_b_^app^) and stability (∆∆G_f_^app^) of the globular heads constituted by three protomers (Protomer–protomer) and of the interaction between globular heads (Trimer–trimer). ∆∆G values are reported as average ± standard deviation of [*n*] possible combinations of WT/mutant complexes.

Number of Mutations [*n*]	∆∆G_b_^app^ (kcal/mol)	∆∆G_b_^app^ C^+^ (kcal/mol)	∆∆G_b_^app^ S * (kcal/mol)	∆∆G_f_^app^ (kcal/mol)	∆∆G_f_^app^ C^+^ (kcal/mol)	∆∆G_f_^app^ S * (kcal/mol)
**Protomer–protomer**						
1 mut [9]	12.2 ± 5.5	−0.43 ± 0.06	12.9 ± 4.7	30.5 ± 1.0	44.7 ± 2.6	−9.0 ± 4.8
2 mut [9]	27.7 ± 6.6	11.4 ± 9.8	16.5 ± 5.2	64.8 ± 1.6	95.3 ± 9.8	−24.1 ± 5.2
3 mut [3]	44.7 ± 1.4	35.3 ± 1.9	9.4 ± 1.2	101.12 ± 0.04	151.8 ± 1.9	−45.5 ± 1.2
**Trimer–trimer**						
1 mut [6]	9.0 ± 9.8	−42.9 ± 5.5	52.4 ± 12.3	26.7 ± 2.8	−90.7 ± 13.6	127.4 ± 25.8
2 mut [15]	18.6 ± 12.4	−83.4 ± 6.7	103.2 ± 15.4	55.6 ± 4.4	−173.7 ± 18.7	248.5 ± 32.7
3 mut [20]	28.6 ± 13.6	−121.1 ± 7.1	151.6 ± 16.9	86.3 ± 4.9	−249.1 ± 20.3	362.9 ± 35.6
4 mut [15]	38.9 ± 13.2	−156.3 ± 7.1	197.8 ± 16.2	118.8 ± 4.7	−316.7 ± 18.1	470.8 ± 34.3
5 mut [6]	49.5 ± 11.2	−190.1 ± 5.6	242.9 ± 13.0	152.7 ± 4.2	−376.7 ± 12.3	572.0 ± 28.4
6 mut [1]	60.0	−221.7	285.9	187.8	−429.0	666.3

^+^ Coulombic contribution to ∆∆G_b_^app^ and ∆∆G_f_^app^; * solvation contribution to ∆∆G_b_^app^ and ∆∆G_f_^app^.

**Table 4:** Phenotypes of *C1QTNF5* associated disorders: adGALCD and LORD.

	adGALCD	LORD
*C1QTNF5*	c.538C>G, p.(Q180E)	c.489C>G, p.(S163R) c.489C>A, p.(S163R) c.556C>T, p.(P186S) c.562C>A, p.(P188W) c.569C>G, p.(S190W) c.646G>T, p.(G216C)
Age at onset symptoms	40 years	40–50 years
Initial functional deficits	Problems with adaption in the dark, night blindness	Problems with adaption in the dark, night blindness
Age at onset of retinal alterations	18–24 years	44–50 years
Area of onset	Peripheral and peripapillary atrophy	Midperipheral, temporal to the macula
Pseudodrusen-like changes	No	Yes
Sub-RPE deposits	No	Yes
Choroidal atrophy	Large and confluent, sharply demarcated	Scalloped beginning temporal of the macula in areas with previous pseudodrusen, irregular borders
Pigmentation	No or minimal	Moderate to marked
Choroidal neovascularization	Not observed	Frequent during progression
Macular edema	May occur	Secondary to choroidal neovascularization
FAF	Large, sharply demarcated areas of absent/severely reduced FAF	Fleck-like irregular or scalloped midperipheral loss bordered by increased FAF, irregular in macular lesions
OCT	No deposits, absence of RPE and photoreceptors in affected areas	Subretinal deposits, irregular photoreceptor loss
ERG	Markedly reduced at age 38, residual or not measurable responses at age >50 years	Normal or well-preserved ERG up to 60–67 years of age
mfERG	Reduced amplitude, normal implicit time in preserved areas, no response in affected areas	Not reported
Anterior segment	Normal	Long anterior lens zonules in most patients

adGALCD: autosomal dominant gyrate atrophy-like choroidal dystrophy, LORD: late-onset retinal degeneration, RPE: retinal pigment epithelium, FAF: fundus autofluorescence, OCT: optical coherence tomography, ERG: full-field electroretinography, mfERG: multifocal ERG.

## Data Availability

The data presented in this study are available on request from the corresponding author. The data are not publicly available due to health system data regulations.

## Abbreviations

adGALCD	Autosomal dominant gyrate atrophy-like choroidal dystrophy
ERG	Electroretinography
FAF	Fundus autofluorescence
LORD	Late onset retinal dystrophy
mfERG	Multifocal electroretinography
OCT	Optical coherence tomography
RPE	Retinal pigment epithelium
WT	Wild-type

## Appendix A

**Table A1:** In silico predictions of different pathogenicity-computation methods for the p.(Q180E) variant.

PolyPhen2	MutationTaster	PROVEAN	SIFT	phyloP	CADD	FATHMM-MKL
Possibly damaging (0.692)	Disease causing (0.999)	Neutral (0.38)	Tolerated (0.254)	9.071	25.00	0.98

**Table A2:** Sequence conservation of the p.(Q180E) variant in orthologous C1QTNF5 protein sequences.

Species	Amino Acid Snippet
*Homo sapiens*	178 FFQFFGGWPKPASLSGGAMVR 198
*Acanthisitta chloris*	178 FFQYYGNWPKPTSLSGGTLVR 198
*Acinonyx jubatus*	92 FFQFFGGWPKPASLSGGAMVR 112
*Ailuropoda melanoleuca*	88 FFQFFGGWPKPASLSGGAMVR 108
*Alligator mississippiensis*	176 FFQFYGNWPKPTSLSGGSLVR 196
*Alligator sinensis*	176 FFQFYGNWPKPTSLSGGSLVR 196
*Anas platyrhynchos*	353 FFQYYGNWPKPTSLSGGALVR 373
*Anolis carolinensis*	180 FFQFYGNWPKPTSLSGGALVR 200
*Antrostomus carolinensis*	64 FFQYYGNWPKPTSLSGGALVR 84
*Aotus nancymaae*	178 FFQFFGGWPKPASLSGGAMVR 198
*Aptenodytes forsteri*	178 FFQYYGNWPKPTSLSGGSLVR 198
*Astyanax mexicanus*	183 YFQFFGNWSKPASLSGGTLMH 203
*Balaenoptera acutorostrata scammoni*	151 FFQFFGGWPKPASLSGGAMVR 171
*Boleophthalmus pectinirostris*	184 YFQFYGNWPKPVSLSGGSLLH 204
*Bos indicus*	178 FFQFFGGWPKPASLSGGAMVR 198
*Bos mutus*	134 FFQFFGGWPKPASLSGGAMVR 154
*Bos taurus*	178 FFQFFGGWPKPASLSGGAMVR 198
*Bubalus bubalis*	178 FFQFFGGWPKPASLSGGAMVR 198
*Calidris pugnax*	178 FFQYYGNWPKPTSLSGGALVR 198
*Callithrix jacchus*	289 FFQFFGGWPKPASLSGGAMVR 309
*Callorhinchus milii*	180 FFQFYGNWTKPVSLSGGSLVH 200
*Calypte anna*	180 FFQYYGNWPKPTSLSGGALVR 200
*Camelus bactrianus*	178 FFQFFGGWPKPASLSGGAMVR 198
*Camelus ferus*	90 FFQFFGGWPKPASLSGGAMVR 110
*Canis lupus*	189 FFQFFGGWPKPASLSGGAMVR 209
*Capra hircus*	178 FFQFFGGWPKPASLSGGAMVR 198
*Castor canadensis*	238 FFQFFGGWPKPASLSGGAMVR 258
*Cavia porcellus*	178 FFQFFGGWPKPASLSGGAMVR 198
*Ceratotherium simum simum*	178 FFQFFGGWPKPASLSGGAMVR 198
*Cercocebus atys*	178 FFQFFGGWPKPASLSGGAMVR 198
*Chaetura pelagica*	150 FFQYYGNWPKPTSLSGGALVR 170
*Charadrius vociferus*	177 FFQYYGNWPKPTSLSGGALVR 197
*Chelonia mydas*	176 FFQFYGNWPKPTSLSGGALVR 196
*Chinchilla lanigera*	178 FFQFFGGWPKPASLSGGTMVR 198
*Chlorocebus sabaeus*	178 FFQFFGGWPKPASLSGGAMVR 198
*Chrysemys picta*	181 FFQFYGNWPKPTSLSGGALVR 201
*Chrysochloris asiatica*	178 FFQFFGGWPKPASLSGGAMVR 198
*Clupea harengus*	183 YFQFFGNWSKPASLSGGTLAH 203
*Columba livia*	178 FFQYYGNWPKPTSLSGGSLVR 198
*Condylura cristata*	276 FFQFFGGWPKPTSLSGGAMVR 296
*Corvus brachyrhynchos*	178 FFQYYGNWPKPTSLSGGTLVR 198
*Coturnix japonica*	182 FFQYYGNWPKPTSLSGGALVR 202
*Cricetulus griseus*	273 FFQFFGGWPKPASLSGGAMVR 293
*Crocodylus porosus*	176 FFQFYGNWPKPASLSGGSLVR 196
*Cuculus canorus*	178 FFQYYGNWPKPTSLSGGALVR 198
*Cynoglossus semilaevis*	187 YFQFYGNWPKPASLSGGSLLH 207
*Cyprinodon variegatus*	185 YFQFYGNWPKPASLSGGSLLH 205
*Cyprinus carpio*	183 YFQIFGNWSKPASLSGGTLVH 203
*Danio rerio*	183 YFQIFGNWSKPASLSGGTLVH 203
*Dasypus novemcinctus*	178 FFQFFGGWPKPASLSGGAMVR 198
*Dipodomys ordii*	130 FFQFFGGWPKPASLSGGAMVR 150
*Egretta garzetta*	178 FFQYYGNWPKPTSLSGGALVR 198
*Elephantulus edwardii*	178 FFQFFGGWPKPASLSGGAMVR 198
*Eptesicus fuscus*	80 FFQVFGGWPKPASLSGGAMVR 100
*Equus asinus*	178 FFQFFGGWPKPASLSGGAMVR 198
*Equus caballus*	178 FFQFFGGWPKPASLSGGAMVR 198
*Equus przewalskii*	60 FFQFFGGWPKPASLSGGAMVR 80
*Erinaceus europaeus*	322 FFQFFGGWPKPTSLSGGAMVR 342
*Esox lucius*	183 YFQFYGNWPKPVSLTGGSLLH 203
*Falco cherrug*	178 FFQYYGNWPKPTSLSGGALVR 198
*Falco peregrinus*	178 FFQYYGNWPKPTSLSGGALVR 198
*Felis catus*	178 FFQFFGGWPKPASLSGGAMVR 198
*Ficedula albicollis*	178 FFQYYGNWPKPTSLSGGTLVR 198
*Fukomys damarensis*	178 FFQFFGGWPKPASLSGGAMVR 198
*Fundulus heteroclitus*	185 YFQYYGNWSKPASLSGGTMLH 205
*Gadus morhua*	245 YFQFYGNWPKPASLSGGTMLH 265
*Galeopterus variegatus*	92 FFQFFGGWPKPASLSGGAMVR 112
*Gallus gallus*	182 FFQYYGNWPKPTSLSGGALVR 202
*Gavialis gangeticus*	176 FFQFYGNWPKPASLSGGSLVR 196
*Geospiza fortis*	178 FFQYYGNWPKPTSLSGGTLVR 198
*Gorilla gorilla*	178 FFQFFGGWPKPASLSGGAMVR 198
*Haliaeetus leucocephalus*	178 FFQYYGNWPKPTSLSGGALVR 198
*Haplochromis burtoni*	185 YFQFYGNWPKPASLSGGSLLH 205
*Heterocephalus glaber*	178 FFQFFGGWPKPASLSGGAMVR 198
*Hippocampus comes*	185 YFQFFGNWPKPVSLSGGSLLH 205
*Hipposideros armiger*	236 FFQFFGGWPKPASLSGGTMVR 256
*Ictalurus punctatus*	183 YFQMFGNWSKPASLSGGTLLH 203
*Ictidomys tridecemlineatus*	178 FFQFFGGWPKPASLSGGAMVR 198
*Jaculus jaculus*	179 FFQFFGGWPKPASLSGGAMVR 199
*Kryptolebias marmoratus*	224 YFQIYGNWSKPASLSGGSLLH 244
*Labrus bergylta*	185 YFQFYGNWPKPASLSGGSLLH 205
*Larimichthys crocea*	185 YFQFYGNWPKPASLSGGSLLH 205
*Lates calcarifer*	185 YFQFYGNWPKPASLSGGSLLH 205
*Latimeria chalumnae*	179 FFQFYGNWPKPSSLSGGTLLH 199
*Lepidothrix coronata*	178 FFQYYGNWPKPTSLSGGTLVR 198
*Lepisosteus oculatus*	183 YFQYYGNWPKPASLSGGSLLH 203
*Leptonychotes weddellii*	153 FFQFFGGWPKPASLSGGAMVR 173
*Leptosomus discolor*	178 FFQYYGNWPKPTSLSGGALVR 198
*Lipotes vexillifer*	178 FFQFFGGWPKPASLSGGAMVR 198
*Loxodonta africana*	178 FFQFFGGWPKPASLSGGAMVR 198
*Macaca fascicularis*	327 FFQFFGGWPKPASLSGGAMVR 347
*Macaca mulatta*	327 FFQFFGGWPKPASLSGGAMVR 347
*Macaca nemestrina*	178 FFQFFGGWPKPASLSGGAMVR 198
*Manacus vitellinus*	178 FFQYYGNWPKPTSLSGGTLVR 198
*Mandrillus leucophaeus*	166 FFQFFGGWPKPASLSGGAMVR 186
*Manis javanica*	178 FFQFFGGWPKPASLSGGAMVR 198
*Marmota marmota marmota*	88 FFQFFGGWPKPASLSGGAMVR 108
*Maylandia zebra*	185 YFQFYGNWPKPASLSGGSLLH 205
*Meleagris gallopavo*	178 FFQYYGNWPKPTSLSGGALVR 198
*Melopsittacus undulatus*	178 FFQYYGNWPKPTSLSGGALVR 198
*Mesitornis unicolor*	182 FFQYYGNWPKPTSLSGGALVR 202
*Mesocricetus auratus*	203 FFQFFGGWPKPASLSGGAMVR 223
*Microcebus murinus*	178 FFQFFGGWPKPASLSGGAMVR 198
*Microtus ochrogaster*	178 FFQFFGGWPKPASLSGGAMVR 198
*Monodelphis domestica*	261 FFQFFGGWPKPASLSGGALVR 281
*Monopterus albus*	185 YFQFYGNWSKPASLSGGSLLH 205
*Mus musculus*	178 FFQYFGGWPKPASLSGGAMVR 198
*Mustela putorius furo*	178 FFQFFGGWPKPASLSGGAMVR 198
*Myotis brandtii*	51 FFQFFGGWPKPASLSGGAMVR 71
*Myotis lucifugus*	120 FFQFFGGWPKPASLSGGAMVR 140
*Nannospalax galili*	178 FFQFFGGWPKPASLSGGAMVR 198
*Nipponia nippon*	178 FFQYYGNWPKPTSLSGGALVR 198
*Nomascus leucogenys*	201 FFQFFGGWPKPASLSGGAMVR 221
*Nothobranchius furzeri*	198 YFQFYGNWPKPASLSGGSLLH 218
*Notothenia coriiceps*	185 YFQYYGNWPKPASLSGGSLLH 205
*Ochotona princeps*	179 FFQFFGGWPKPASLSGGAMVR 199
*Octodon degus*	178 FFQFFGGWPKPASLSGGAMVR 198
*Odobenus rosmarus divergens*	178 FFQFFGGWPKPASLSGGAMVR 198
*Oncorhynchus kisutch*	186 YFQFYGNWPKPASLSGGSLLH 206
*Oncorhynchus mykiss*	184 YFQFYGNWPKPASLSGGSLLH 204
*Orcinus orca*	178 FFQFFGGWPKPASLSGGTMVR 198
*Oreochromis niloticus*	185 YFQFYGNWPKPASLSGGSLLH 205
*Ornithorhynchus anatinus*	181 FFQFFGGWPKPASLSGGALVR 201
*Orycteropus afer afer*	178 FFQFFGGWPKPASLSGGAMVR 198
*Oryctolagus cuniculus*	178 FFQFFGGWPKPASLSGGAMVR 198
*Oryzias latipes*	183 YFQFYGSWPKPASLSGGSLLH 203
*Otolemur garnettii*	178 FFQFFGGWPKPASLSGGAMVR 198
*Ovis aries*	178 FFQFFGGWPKPASLSGGAMVR 198
*Pan paniscus*	178 FFQFFGGWPKPASLSGGAMVR 198
*Pan troglodytes*	178 FFQFFGGWPKPASLSGGAMVR 198
*Panthera pardus*	178 FFQFFGGWPKPASLSGGAMVR 198
*Panthera tigris altaica*	79 FFQFFGGWPKPASLSGGAMVR 99
*Pantholops hodgsonii*	91 FFQFFGGWPKPASLSGGAMVR 111
*Papio anubis*	178 FFQFFGGWPKPASLSGGAMVR 198
*Paralichthys olivaceus*	185 YFQFYGNWPKPGSLSGGSLLH 205
*Parus major*	178 FFQYYGNWPKPTSLSGGSLVR 198
*Pelodiscus sinensis*	176 FFQFYGNWPKPTSLSGGALVR 196
*Peromyscus maniculatus bairdii*	273 FFQFFGGWPKPASLSGGAMVR 293
*Phascolarctos cinereus*	178 FFQFFGGWPKPASLSGGALVR 198
*Physeter catodon*	234 FFQFFGGWPKPASLSGGAMVR 254
*Picoides pubescens*	182 FFQYYGNWPKPTSLSGGALVR 202
*Poecilia formosa*	185 YFQFYGNWPKPASLSGGSLLH 205
*Poecilia latipinna*	185 YFQFYGNWPKPASLSGGSLLH 205
*Poecilia reticulata*	185 YFQFYGNWPKPASLSGGSLLH 205
*Pogona vitticeps*	182 FFQFYGNWPKPTSLSGGVLVR 202
*Pongo abelii*	178 FFQFFGGWPKPASLSGGAMVR 198
*Propithecus coquereli*	178 FFQFFGGWPKPASLSGGAMVR 198
*Pseudopodoces humilis*	178 FFQYYGNWPKPTSLSGGSLVR 198
*Pteropus alecto*	225 FFQFFGGWPKPASLSGGAMVR 245
*Pteropus vampyrus*	225 FFQFFGGWPKPASLSGGAMVR 245
*Pundamilia nyererei*	185 YFQFYGNWPKPASLSGGSLLH 205
*Pygocentrus nattereri*	193 YFQIFGNWSKPASLSGGTLLH 213
*Python bivittatus*	189 FFQFYGNWPKPTSLSGGSLVR 209
*Rattus norvegicus*	178 FFQFFGGWPKPASLSGGAMVR 198
*Rhinolophus sinicus*	178 FFQFFGGWPKPASLSGGTMVR 198
*Rhinopithecus bieti*	178 FFQFFGGWPKPASLSGGAMVR 198
*Rhinopithecus roxellana*	178 FFQFFGGWPKPASLSGGAMVR 198
*Rousettus aegyptiacus*	178 FFQFFGGWPKPASLSGGAMVR 198
*Saimiri boliviensis*	178 FFQFFGGWPKPASLSGGAMVR 198
*Salmo salar*	186 YFQFYGNWPKPASLSGGSLLH 206
*Sarcophilus harrisii*	154 FFQFFGGWPKPASLSGGALVR 174
*Scleropages formosus*	182 YFQFYANWPKPASLSGGSLLH 202
*Sorex araneus*	178 FFQFFGGWPKPASLSGGTMVR 198
*Stegastes partitus*	185 YFQFYGNWPKPASLSGGSLLH 205
*Sturnus vulgaris*	178 FFQYYGNWPKPTSLSGGTLVR 198
*Sus scrofa*	195 FFQFFGGWPKPASLSGGAMVR 215
*Taeniopygia guttata*	251 FFQYYGNWPKPTSLSGGTLVR 271
*Takifugu rubripes*	231 YFQFYGNWPKPVSLSGSSLLH 251
*Tauraco erythrolophus*	168 FFQYYGNWPKPTSLSGGALVR 188
*Tinamus guttatus*	182 FFQYYGNWPKPTSLSGGALVR 202
*Trichechus manatus latirostris*	178 FFQFFGGWPKPASLSGGAMVR 198
*Tupaia chinensis*	178 FFQFFGGWPKPASLSGGAMVR 198
*Ursus maritimus*	55 FFQFFGGWPKPASLSGGAMVR 75
*Vicugna pacos*	131 FFQFFGGWPKPASLSGGAMVR 151
*Xenopus tropicalis*	179 FFQL-GDAKKPVGLCGGAALR 198
*Xiphophorus maculatus*	185 YFQFYGNWPKPASLSGGSLLH 205

Orthologous sequences were obtained from NCBI and aligned using ClustalW. The conserved glutamine residue is shown in red.
